# 5-Dodecanolide, a Compound Isolated from Pig Lard, Presents Powerful Anti-Inflammatory Properties

**DOI:** 10.3390/molecules26237363

**Published:** 2021-12-04

**Authors:** Xavier Capó, Miquel Martorell, Josep A. Tur, Antoni Sureda, Antoni Pons

**Affiliations:** 1 Research Group on Community Nutrition and Oxidative Stress, Health Research Institute of the Balearic Islands (IdISBa), 07120 Palma, Spain; xavier.capo@uib.es (X.C.); pep.tur@uib.es (J.A.T.); antonio.sureda@uib.es (A.S.); 2 Research Group on Community Nutrition and Oxidative Stress, Laboratory of Physical Activity Sciences, University of the Balearic Islands, 07122 Palma, Spain; 3 Department of Nutrition and Dietetics, Faculty of Pharmacy, Centre for Healthy Living, University of Concepcion, Concepcion 4070386, Chile; martorellpons@gmail.com; 4 CIBEROBN (Physiopathology of Obesity and Nutrition CB12/03/30038), Carlos III Health Institute, 28029 Madrid, Spain

**Keywords:** pig lard, inflammation, 5-dodecanolide, lipid mediators

## Abstract

Background: Pork lard (PL) is traditionally used as an anti-inflammatory agent. We propose to demonstrate the anti-inflammatory properties of PL, and elucidate which compounds could be responsible for the anti-inflammatory effects. Methods: The anti-inflammatory effects of PL were tested in a rat model of zymosan-induced hind paw inflammation. Further, the hydroalcoholic extract from PL was obtained, the composition analyzed, and the anti-inflammatory activity of the extracts and isolated components assayed using immune cells stimulated with lipopolysaccharide (LPS). Results: Applying the ointment on the inflamed rat feet reduced the foot diameter, foot weight, and activities of antioxidant enzymes and inflammatory markers of circulating neutrophils. The main components of the hydroalcoholic extract were 5-dodecanolide, oleamide, hexadecanoic acid, 9-octadecenoic acid, hexadecanamide, and resolvin D1. Conclusions: PL reduces the immune response in an animal model stimulated with zymosan. Hydroalcoholic PL extract and its components (5-Dodecanolide, Oleamide, and Resolvin D1) exerted an anti-inflammatory effect on LPS-stimulated neutrophils and peripheral mononuclear cells reducing the capability to produce TNFα, as well as the activities of antioxidant and pro-inflammatory enzymes. These effects are attributable to 5-dodecanolide, although the effects of this compound alone do not reach the magnitude of the anti-inflammatory effects observed by the complete hydroalcoholic extract.

## 1. Introduction

Inflammation is a tissue process that consists of a series of molecular, cellular, and vascular phenomena with a defensive purpose against physical, chemical, or biological aggressions [1,2]. The inflammatory process is an immediate and nonspecific response that can facilitate the subsequent development of a specific response [3]. Furthermore, the process is usually focused on a definite area, although there may be exceptions, as in the case of systemic inflammation [4]. Inflammation is characterized by the migration of immune cells toward the inflammatory focus. As a consequence of inflammation, there is dilation and an increase in the permeability of the blood vessels near the damaged site in order to facilitate the arrival and trans-endothelial migration of leukocytes to the inflamed area, in addition to facilitating the arrival of other molecular mediators [1]. The ultimate goal of inflammation is to eliminate or inhibit the causative agent of infection, prevent cell damage, and allow the body to recover normal conditions, restoring the functionality of the affected tissue or organ [2]. The resolution of acute inflammation can also be mediated by pro-resolving lipid mediators, which include the lipoxin, resolvin, protectin and maresin families derived from the oxidative pathways of omega-3 essential fatty acids [5].

Skin aging is a multifaceted biological phenomenon consequence of both intrinsic (or chronological) and extrinsic factors, including exposure to UV rays (or photo-aging), air pollution, and smoking [6]. Intrinsic or innate aging is a genetically determined process associated with cellular aging as a result of progressive and inevitable dermal atrophy. However, both aging processes are directly related to oxidative stress and inflammation [7,8]. Skin damage is often associated with the overproduction of reactive oxygen species (ROS). In this sense, it is evidenced that ROS activate mitogen-activated protein kinases (MAPK) and the nuclear transcription factor kappa B (NFκB) signaling pathways [9]. NFκB plays a central role in many cellular processes, including immune, inflammatory response, and cellular adhesion [10]. For example, as a consequence of NFκB activation, the expression of several pro-inflammatory mediators, including cyclooxygenase 2 (COX-2), is induced [11].

Several studies have evidenced a strong anti-inflammatory effect of several oxidized lipids [12,13]. Arachidonic acid oxidation products, such as 1-palmitoyl-2-arachidonoyl-sn-glycerol-3-phosphatidylcholine (PAPC), have been reported to be able to reduce the pro-inflammatory effects of lipopolysaccharide (LPS) by blocking the LPS-Toll-like receptor 4 (TLR4)-NFκB inflammatory cascade and reducing the production of inflammatory mediators, including the tumor necrosis factor alpha (TNFα) [12]. In fact, PAPC and products derived from its oxidation have been found to accumulate at sites of inflammation [14]. These compounds are also capable of reducing inflammation in lung infections by lowering interleukin (IL)-1β and IL-6 levels, ameliorating neutrophil infiltration, and inhibiting inducible nitric oxide synthase (iNOS) expression [13,15]. In addition, these compounds induce the production of pro-resolution and anti-inflammatory mediators, such as lipoxin A4 (LXA4) in mice intratracheally injected with LPS [16].

Traditionally, pork lard (PL), obtained after prolonged cooking of pork white adipose tissue, had been used as an anti-inflammatory treatment against strokes and acute inflammatory processes [17,18,19,20], but without knowing the components that may be responsible for these effects. During the manufacturing process, lipids suffer oxidative reactions that generate several oxidation products. However, several studies have shown the anti-inflammatory effects of topically applied lard, usually in combination with other ingredients. In these studies, lard is used in combination with components such as honey, olive oil, camphor, bicarbonate, or rosemary as a remedy for inflammatory processes, anti-erythematosus, eczema, atopic dermatitis, hair loss, varicose veins, joint pain, and cramps [17,18,19]. Furthermore, it has been evidenced that a lard-based high-fat diet increases secretory leukocyte protease inhibitor expression and attenuates the inflammatory response of acute lung injury in endotoxemic rats [21]. These anti-inflammatory properties could be related to the presence of oxidized lipids in pork lard [22], derived from the oxidative process at high temperatures, which allows one to obtain pork lard from the pig adipose tissues. Due to the lack of specific information on the possible compounds responsible for the anti-inflammatory effects, the aim of the present study is to demonstrate the anti-inflammatory properties of pork lard, and to elucidate which compounds could be responsible for its anti-inflammatory effects.

## 2. Results

### 2.1. Effects of PL in In Vivo Animal Model

**Figure 1:**
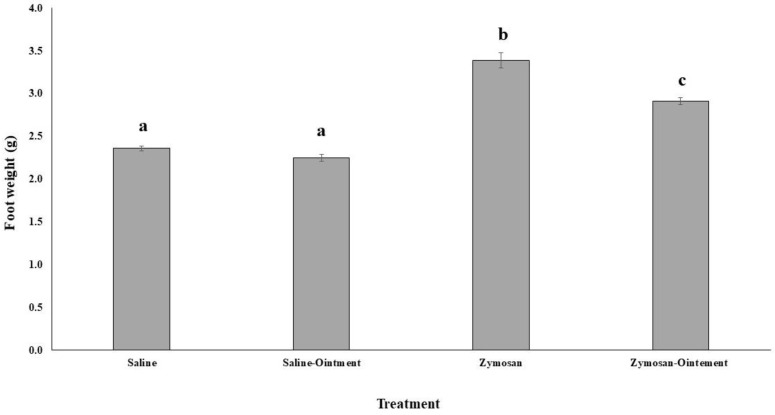
Effects of pig lard treatment in foot weight after inflammatory stimulation. Statistical analysis: One-way ANOVA test. Different letters indicate significant differences (*p* < 0.05).

**Figure 2:**
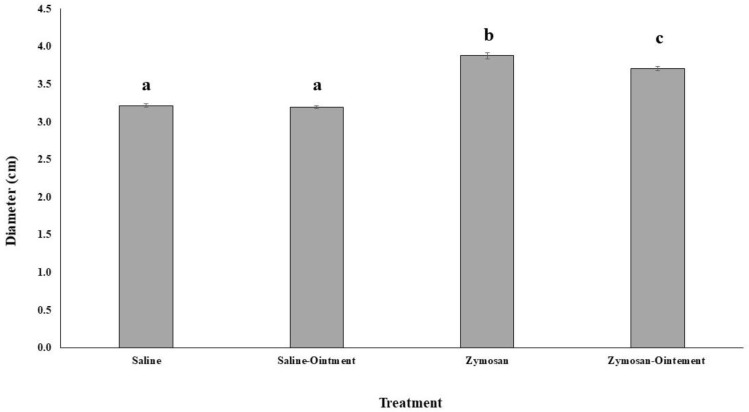
Effects of pig lard treatment in leg diameter after inflammatory stimulation. Statistical analysis: One-way ANOVA test. Different letters indicate significant differences (*p* < 0.05).

Foot diameter and total foot weight were measured as indicators of the inflammation degree as a consequence of a zymosan subplantar injection. Injection of plasma-activated zymosan in rat paws evoked an inflammatory reaction with a maximum oedema 1 h after administration (Figure 1). The treatment of inflamed feet with PL significantly reduced the foot diameter in comparison to inflamed feet treated with vaseline; this difference was significant after 3 h and 4 h of treatment with PL. After 4 h of foot injections with the zymosan or saline and treated with PL, animals were sacrificed and leg weight was measured (Figure 2). Animals injected with zymosan presented a significantly higher foot weight than animals injected with saline buffer. The treatment with PL did not influence the foot weight after saline injection, but it significantly reduced the increased foot weight after the zymosan injection-induced inflammation.

### 2.2. Antioxidant and Proinflammatory Markers in In Vivo Animal Model

The activities of the antioxidant enzymes catalase (CAT), glutathione peroxidase (GPx), and the pro-inflammatory myeloperoxidase (MPO) were measured in subplantar tissue and in neutrophils. The levels malondialdehyde (MDA), carbonyl protein derivates and nitrite, measured in subplantar tissues homogenates, were used as indicators of oxidative damage and inflammation (Table 1). No effects were evidenced by the injection of zymosan or by the application of PL in the CAT and GPx activities. MPO activity significantly increased as a consequence of the zymosan injection, but the treatment of inflamed feet with PL maintained significantly higher MPO activity. No effects of either zymosan or PL treatment were observed in MDA levels. The zymosan injection induced an increase in carbonyl protein derivates, but no effects of the PL treatment were observed. On the other hand, nitrite levels significantly increased as a consequence of the zymosan injection, and this increase was significantly reduced in PL-treated animals.

Smearing the feet with PL ointment affects the activities of antioxidant enzymes and inflammatory markers in circulating neutrophils (Table 1). CAT activity was significantly increased after zymosan injection, and its activity was significantly reduced after PL treatment reached control levels. Similarly, MPO activity was significantly increased after the zymosan injection and this increase was significantly reduced to values similar to a non-inflamed foot after PL treatment. No effects of zymosan or PL treatment were evidenced in GPx activity in neutrophils.

### 2.3. PL Hydroalcoholic Extract Composition

The composition of the PL hydroalcoholic extract is shown in Table 2. The gas chromatography-mass spectrometry analysis of the PL hydroalcoholic extract evidenced the presence of several free mono unsaturated fatty acids, such as 9-octadecenoic, 6-octadecenoic, and 7-hexadecenoic, which represent about 77% of the components of hydroalcoholic extract. It also contains polyunsaturated free fatty acids, such as 5,8,11,14-eicosatetraenoic, representing about 5.8%, 11,14-octadecadienoic, representing about 5.8%, and low content 9,12-octadecenoic, representing about 0.3%. Other compounds probably present as result of fatty acid transformation during the production of PL from the pork adipose tissues were 5-dodecanolide (5-dodecanolide, about 4.5%), hexadecanamide, (about 1%), and octadecanamide (oleamide, about 5%). The presence of Resolvin D1 (2.9 mg/g) was also detected using a specific immunological detection analysis.

### 2.4. Effects of PL Hydroalcoholic Extract in Ex Vivo Immune Cell Model

**Figure 3:**
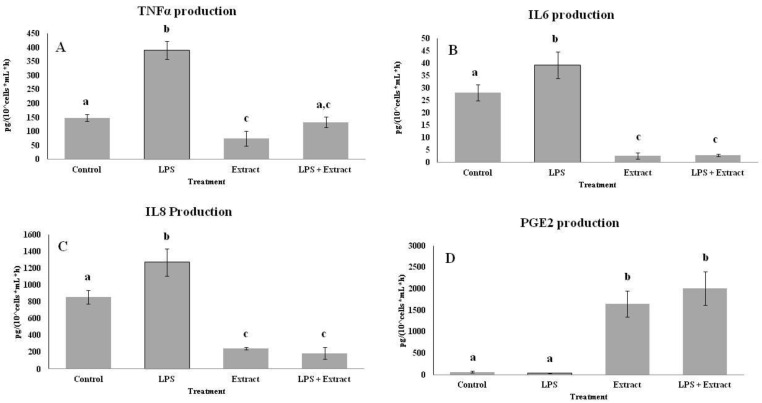
Cytokines and prostaglandin production rate by PBMCs stimulated with LPS in presence or absence of pig lard hydroalcoholic extract. TNFα production (**A**); IL6 production (**B**); IL8 production (**C**); PGE2 production (**D**). Statistical analysis: One-way ANOVA test. Different letters indicate significant differences between treatments (*p* < 0.05).

The rate of TNF-α, IL-6, IL-8, and PGE-1 production by peripheral blood mononuclear cells (PBMCs) stimulated with LPS were measured as inflammatory markers to evidence a possible anti-inflammatory effect of the PL hydroalcoholic extract (Figure 3). TNF-α (Figure 3A), IL-6 (Figure 3B), and IL-8 (Figure 3C) PBMCs production was increased in LPS-activated groups. A reduction in the production of the three cytokines was observed in the hydroalcoholic PL extract group in comparison with the control group. The combination of LPS with the hydroalcoholic extract reduced the pro-inflammatory effects of LPS. PGE-1 (Figure 3D) PBMCs production was not modulated by LPS activation. However, the hydroalcoholic extract causes a significant increase in PGE-1 levels both alone and in combination with LPS.

### 2.5. Antioxidant and Anti-Inflammatory Properties of PL Components

**Figure 4:**
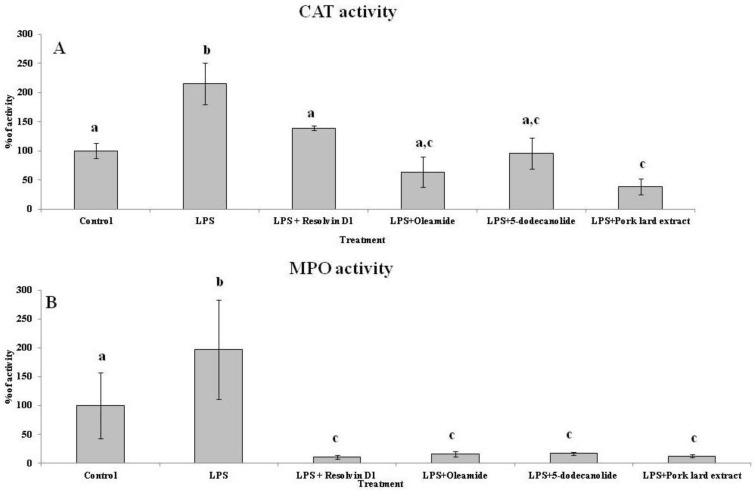
Antioxidant and anti-inflammatory effects of pork lard hydroalcoholic extract compounds neutrophils LPS-stimulated. CAT (**A**); MPO (**B**). Statistical analysis: One-way ANOVA test. Different letters indicate significant differences between the treatmnets (*p* < 0.05).

The antioxidant and anti-inflammatory effects of several compounds present in the PL hydroalcoholic extract were assayed in ex vivo neutrophils by evaluating the catalase and myeloperoxidase activities in LPS-stimulated cells (Figure 4). The CAT (Figure 4A) and MPO (Figure 4B) activities in the neutrophil from the control cultures were used as reference values (100%). The neutrophil stimulation with LPS significantly duplicated the CAT and MPO activities whereas the presence of PL hydroalcoholic extract significantly reduced these activities until control values for CAT were significantly lower than the control for MPO. Resolvin D1, oleamide and 5-dodecanolide, all three present in the hydroalcoholic PL extract, reduced the MPO activities of the LPS-stimulated neutrophils until values were significantly lower than the non-LPS-stimulated control neutrophils. These compounds also reduced the CAT activity of LPS-stimulated neutrophils to the non-stimulated control values.

**Figure 5:**
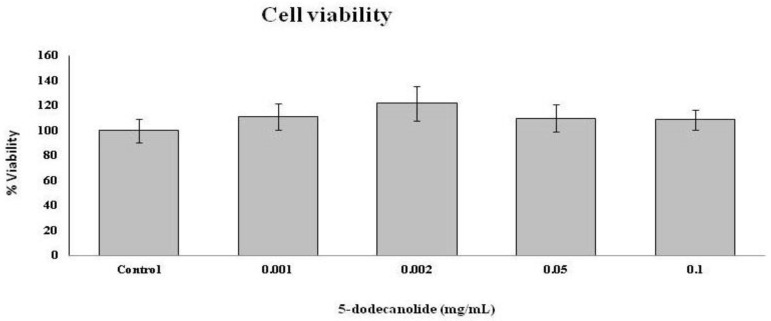
Effects of 0, 0.001, 0.005, 0.01, 0.02, 0.05, and 0.1 mg/mL of 5-dodecanolide in cell viability. Statistical analysis: One-way ANOVA test. Different letters indicate significant differences (*p* < 0.05).

Treatment with 5-dodecanolie at 0.1 mg/mL did not cause cell mortality, nor at lower concentrations (Figure 5)

**Figure 6:**
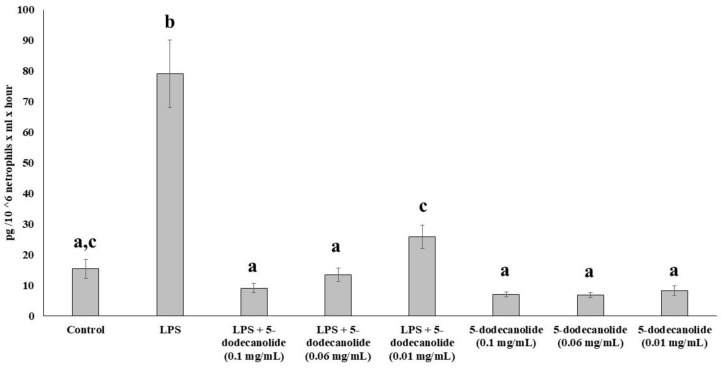
Effects of 5-dodecanolide in neutrophil TNFα production. Statistical analysis: One-way ANOVA test. Different letters indicate significant differences between treatments (*p* < 0.05).

The dose-dependent anti-inflammatory properties of 5-dodecanolide were evaluated using the ex vivo neutrophil model. Isolated neutrophils were stimulated with LPS in the presence or in the absence of different concentrations of 5-dodecanolide, and the production rate of TNF-α was used as an inflammatory marker (Figure 6). LPS significantly increased the TNF-α production by neutrophils about five times, with respect to the production rate of non-stimulated neutrophils. The different concentrations of 5-dodecanolide tested (0.1 mg/mL, 0.06 mg/mL, and 0.01 mg mL) did not influence the basal production of TNF-α by non-stimulated neutrophils. The three tested concentrations inhibited TNF-α production in LPS-stimulated neutrophils returning to the control values in a concentration-dependent form. The main inhibitory effects were evidenced at 0.1 mg/mL of 5-dodecanolide whereas the lowest inhibitory effects were observed at 0.01 mg/mL of 5-dodecanolide.

## 3. Discussion

PL, a food obtained after prolonged cooking of the white adipose tissue, has been traditionally used to treat blows and inflammatory processes. The anti-inflammatory effects of PL have also been evidenced by its ability to attenuate the inflammatory response of acute lung injury in endotoxemic rats [20]. We have evidenced that PL administered topically significantly decreased the inflammation induced by zymosan injection in rats. In addition, we also observed a reduction in the diameter of inflammation focus and a greater recovery after the usage of PL as an ointment. The anti-inflammatory actions of PL are parallel to changes in oxidative stress and inflammation markers in circulating neutrophils and in the inflamed tissue. We evidenced a reduction in nitrite levels in the inflamed tissue as a consequence of the treatment with PL. Nitrite levels are an indicator of nitric oxide production, an important inflammatory mediator; high concentrations of nitrite are often related to vasodilation and inflammation [22]. No effects of PL on antioxidant enzymes or in oxidative damage markers in the inflamed zone were evidenced as a consequence of the PL treatment. However, the CAT and MPO activities in circulating neutrophils are influenced by zymosan treatment and also by PL. CAT and MPO activities have previously been used as markers of inflammation [23]. We evidenced an increase in CAT and MPO activities in circulating neutrophils after zymosan treatment. However, the treatment with PL caused a significant reduction in both enzyme activities. Previous studies have reported an increase CAT activity in neutrophils stimulated with PMA [23]. Similarly, the activity of MPO, a pro-inflammatory enzyme secreted by neutrophil degranulation that produces hypochlorous acid with antimicrobial activity [24], was also increased in circulating neutrophils as a result of zymosan-induced inflammation. It is evidenced that an excessive MPO activity is related to an increase in oxidative damage and inflammation [23,24]. PL treatment reduced neutrophil MPO activity in rats treated with zymosan. In accordance, we can state that treatment with PL reduces the inflammation induced by zymosan in the hind paws of rats by modulating the synthesis of nitric oxide in the inflamed area and the antioxidant and inflammatory capacities of circulating neutrophils.

The components of PL responsible for these anti-inflammatory effects and also the mechanisms of action involved are still unknown; however, a molecular basis for the negative regulation of inflammation by lipid peroxidation products has been proposed [25]. It is described that some products from enzymatic and non-enzymatic oxidation of fatty acids, as well as oxidized phospholipid species, could regulate the inflammatory response [12,25]. It has been evidenced that the products of the oxidative modification of phospholipid 1-palmitoyl-2-arachidonoyl-sn-glycero-3-phosphocholine (PAPC) present a powerful anti-inflammatory effect [25]. Oxidative products of arachidonic acid inhibit TNF-α production in cells stimulated with LPS [13]. A synthetic oxidized phospholipid derivative alleviates endotoxin-induced lung injury and inhibits the development of pro-inflammatory T helper 1 cells (Th) [25]. These potential anti-inflammatory compounds of PL could be extracted using a mixture of methanol to water in adequate proportions, to ensure the extraction of free oxidized fatty acids and related derivatives. The hydroalcoholic extract from PL could contain oxidative products of fatty acids with potential anti-inflammatory properties.

The anti-inflammatory properties of the hydroalcoholic extract obtained from PL were tested in human PBMCs from healthy patients. LPS is often used as inflammatory response inductor, inasmuch as to induce the production of several cytokines, such as both Th1 (such as IFNγ, TNFα, and IL8) and Th2-type cytokines (such as IL4) [26,27]. In this sense, we have evidenced an important increase in IL6, IL8, and in TNFα production in PBMCs stimulated with LPS. However, the hydroalcoholic extract obtained from PL significantly ameliorated this production of pro-inflammatory cytokines. These results suggest that some compounds present in the hydroalcoholic extract exert strong anti-inflammatory effects. In fact, it has been evidenced that products from the oxidation of lipids have anti-inflammatory effects [12,13]. In addition, we also evidenced that the hydroalcoholic extract induces an increase in PGE1 production. PGEs are lipid mediators synthesized through the oxidation of AA [28]. PGE1 has powerful vasodilatory effects, with the ability to reduce platelet and immune cell aggregation, as well as to reduce edema formation and exert anti-inflammatory effects [5,29,30,31].

We analyzed the composition of the hydroalcoholic extract obtained from PL in order to determine which compounds could be the responsible for its anti-inflammatory effects. In this sense, we detected several compounds from the fatty acids oxidation, such as 5-dodecanolide, oleamide, hexadecanoic acid, 9-octadecenoic acid, 6-octadecenoic acid, 9,12-octadecadienoic acid, octadecanoic acid, 5,8,11,14-eicosatetraenoic acid, 7-hexadecenoic acid, 11 acid, 14-octadecadienoic, and hexadecanamide, which were not present in white adipose tissue. Moreover, other compounds, such as resolvin D1, increase their concentration during the manufacturing process of PL present in the white adipose tissue. Several compounds detected in the PL hydroalcoholic extract have anti-inflammatory properties. Oleamide suppresses the lipopolysaccharide-induced expression of iNOS and COX-2 through the inhibition of NFkβ activation in BV2 murine microglial cells [32,33]. The 9-octadecenoic reduces the pro-inflammatory gene expression in LPS-stimulated PBMCs [34]. Compounds from the oxidation of eicosapentaenoic and docosahexaenoic acids, such as resolvin D1, maresins, and protectins, are able to reduce the production of pro-inflammatory cytokines [5,34,35,36]. The anti-inflammatory properties of PL extract could be related with the presence of resolvin D1, oleamide, and 9-octadecenoic, but we also investigated the possible anti-inflammatory effect of other PL components, such as 5-dodecanolide.

The anti-inflammatory properties of isolated resolvin D1, 5-dodecanolide, and oleamide were assayed in neutrophils stimulated with LPS using concentrations equivalent to those found in hydroalcoholic extract. Neutrophils play an important role in the development of inflammation, in immune regulation, and in tissue reparation [1,35]. The phagocytic neutrophil function involves an oxidative burst carried out by the enzyme NADPH-oxidase and the secretion of lysosomal enzymes, such as proteases, phospholipases, and glycosidases, among others [34]. Neutrophils are the first cells mobilized against tissue damage or infection, causing an inflammatory response, which is characterized by the release of the content of their granules to the external environment and in an oxidative burst consisting of the production of large amounts of hydrogen peroxide and hypochlorite with high oxygen consumption [37,38]. LPS stimulation increased CAT and MPO activities in the culture medium. The addition of the hydroalcoholic extract, oleamide, and 5-dodecanolide significantly reduced the CAT activity in the extracellular medium, although it should be noted that the greatest decrease was observed after treatment with the complete hydroalcoholic extract. MPO activity in the extracellular medium significantly decreased with the addition of the three pure compounds tested, all of them presenting effects of similar magnitude to those observed with the hydroalcoholic extract.

5-dodecanolide is evidenced as a new anti-inflammatory molecule present in the PL extract. The effects of different concentrations of 5-dodecanolide on the TNF-α production capacity of neutrophils stimulated with LPS were analyzed. The treatment with LPS significantly increased the production of TNF-α by neutrophils; this increase was attenuated by the presence of 5-dodecanolide in the culture medium. The inhibitory effects of 5-dodecanolide on TNF-α production are dose-dependent, with greater inhibition being observed at higher concentrations of 5-dodecanolide. The 5-dodecanolide, also known as 5-dodecalactone or dodecan-5-olide, belongs to the class of delta valerolactones; thus, 5-dodecanolide is considered to be a fatty ester lipid molecule, which could be a new anti-inflammatory for topical use. Outside of the human body, 5-dodecanolide has been detected, but not quantified in, several different foods, such as alcoholic beverages, fruits, herbs and spices, milk and milk products, and now in PL. The use of 5-dodecanolide to attenuate skin aging could be considered because of its relevant associated anti-inflammatory actions. A hazard evaluation of 5-dodecanolide performed by laboratory studies with rats, rabbits, and guinea pigs showed mild skin but not ocular irritation, absence of photochemical irritation in rabbits, and no sensitization reactions in guinea pigs, and did not demonstrate acute toxicity after ingestion [39].

## 4. Materials and Methods

### 4.1. Animals and Zymosan-Induced Hind Paw Inflammation

Forty male Sprague–Dawley rats of 200–220 g were purchased from Charles River Laboratories (Barcelona, Spain) and acclimatized to the laboratory conditions for 1 week. Animals were housed individually and maintained in cages under controlled environmental conditions (20 ± 2 °C; 70% humidity, and 12 h light/dark cycle, lights on at 08:00), with free access to standard food (Panlab A04, Panlab S.L.U., Barcelona, Spain) and tap water throughout the experimental period. All efforts were made to minimize animal suffering and to reduce the number of animals used. Animal studies were performed during the light period and in accordance with the European Convention for the Protection of Vertebrate Animals used for Experimental and other Scientific Purposes (Directive 86/609/EEC) and approved by the Bioethical Committee of the University (approval file number 2019/14/AEPX). The animals were anesthetized by intraperitoneal injection of 50 mg/kg of sodium pentobarbital. The edema was induced into the right hind paw by a single subplantar injection (0.1 mL) containing 1% zymosan dissolved in saline, whereas the control groups were injected only with saline. The animals were divided into four groups of ten animals (two groups treated with zymosan and two control-saline groups). One group of zymosan-injected and another of saline-injected animals were topically treated with the pork lard (0.5 g) from one hour after the injections. The other two groups of animals were treated with Vaseline in the same conditions as the treatment with the lard ointment and were used as control. Foot diameter was determined in all animals at time 0 (before injection) and after 1, 2, 3, and 4 h after the injection. This process was performed following the process previously described [40].

### 4.2. Animal Sample Processing

Rats were sacrificed by decapitation 4 h after treatment and the hind paws were cut off at the levels of the calcaneus bone and weighed in an analytical balance. Paw tissues (1 g) were homogenized in a potassium phosphate buffer 50 mM, pH 7, containing 0.5% hexa-decyl-trimethyl-ammonium bromide, and centrifuged for 30 min at 8000× *g* at 4 °C. An aliquot of the supernatant was used for biochemical assays.

Blood samples were collected in EDTA tubes and neutrophils were purified. Briefly, 5 mL blood samples diluted (1:1) in sterile phosphate-buffered saline (PBS), pH 7.4, layered on 4 mL Ficoll-Paque PLUS reagent (GE Healthcare^®^, Chicago, IL, USA), and centrifuged at 900× *g* for 30 min at room temperature. After centrifugation, the superior mononuclear rich layer was discarded, and red blood cells were separated from the neutrophils by the addition of 10 mL dextran saline 6% for 30 min at room temperature. The neutrophil phase at the bottom was washed first with ammonium chloride and then with PBS, pH 7.4, and centrifuged at 750× *g* for 10 min at 4 °C. Finally, the neutrophils were lysed with distilled water and stored at −80 °C until they were used for biochemical assays.

All results from paw tissues were corrected using the level of protein contents in the samples. Proteins were measured with the Biorad Protein Assay^®^ (Biorad, Hercules, USA) using bovine serum albumin as the standard. DNA from the neutrophils was determined by a fluorimetric method based on the reaction between the DNA and diaminobenzoic acid [41]. The DNA quantified in these samples was used to correct neutrophil enzymatic activities. Unless otherwise specified, all materials used were provided by Sigma (Darmstadt, Germany). All determinations were performed in duplicate.

### 4.3. Enzymatic Activities

Catalase (CAT) and glutathione peroxidase (GPx) activities as antioxidant enzymes and the myeloperoxidase (MPO) activity as the inflammation marker were analyzed both in neutrophils and in subplantar tissue homogenate. CAT was measured following the method described by Aebi based on the decomposition of hydrogen peroxide [42]. GPx was determined following an adaptation of the method described by Flohe and Gunzler [43]. MPO activity was measured following the method described by Capeillère [44]. All antioxidant enzyme activities were determined with a Shimadzu UV-2100 spectrophotometer at 37 °C.

### 4.4. Oxidative Damage Markers

Malondialdehyde (MDA) and protein carbonyl derivates were analyzed as markers of oxidative damage. MDA was measured by a colorimetric assay for MDA determination, based on the reaction of MDA with a chromogenic reagent to yield a stable chromophore with maximal absorbance at 586 nm. Briefly, samples or standards were placed in glass tubes containing n-methyl-2-phenylindole (10.3 mM) in acetonitrile:methanol (3:1). HCl 12N was added, and the samples were incubated for 1 h at 45 °C. Absorbance was measured at 586 nm. Carbonyl protein derivates were measured in subplantar tissue homogenate supernatants by an adaptation of the method Levine et al. (1994) [45]. Samples were deproteinised with metaphosphoric acid. Precipitates were resuspended with 2,4-dinitrophenylhydrazine (DNPH) 10 mM, and incubated for 60 min at 37 °C. Then, samples were precipitated with 20% trichloroacetic acid, and centrifuged for 10 min at 1000× *g* and 4 °C. The precipitate was washed twice with ethanol:ethyl acetate (1:1). Precipitated were resuspended with guanidine 6 M in phosphate buffer 2 mM, pH 2.3. The concentration of carbonyl groups was calculated from the absorbance at 340 nm using the value of 22,000 M^−1^ cm^−1^ for the molar absorption of aliphatic DNPH derivatives. Samples were analyzed against a blank of guanidine solution.

### 4.5. Nitrite Levels

Nitrite levels were determined both in neutrophils and in subplantar tissue homogenates by the acidic Griess reaction using a spectrophotometric method. Samples were deproteinized with acetone and kept overnight at −20 °C. Then samples were centrifuged for 10 min at 15,000× *g* at 4 °C, and supernatants were recovered. A 96-well plate was loaded with the samples or nitrite standard solutions (100 µL) in duplicate.of A total 50 µL of sulphanilamide (2% *w*/*v*) in 5% HCl was added to each well, and 50 µL of *N*-(1-naphthyl)-ethylenediamine (0.1% *w*/*v*) in water was later added. After an incubation of 30 min, the absorbance was measured at 540 nm [46].

### 4.6. Ex Vivo Immune Cell Model of Inflammation

#### 4.6.1. Pork Lard Hydroalcoholic Extract Obtention

The hydroalcoholic extract of the PL was obtained by melting 1 g of the PL at 60 °C and adding 5 mL to a mixture of methanol to water (65:35, *v*:*v*) and a 50 µL of 1 M HCl. The mixture was agitated vigorously with mechanical assistance and the phases were separated by centrifugation. The anti-inflammatory principles were, then, purified by means of a solid phase extraction, which was performed using a C18 column (Sep-Pak^®^ Vac 12cc (2 g)) (Supelco Co., St. Louis, MO, USA). The column was washed with 10 mL of water and 6 mL of hexane. Finally, the anti-inflammatory compounds were eluted with 8 mL of methyl-formate. The final eluted was evaporated under a nitrogen stream at 55 °C. The dry residue was dissolved in RPMI1640 culture media at 1 mg/mL. This process was performed in quintuplicate.

#### 4.6.2. Hydroalcoholic Extract Composition

The dry residue of the hydroalcoholic extract of PL obtained from 10 g, according to the described procedure, was dissolved in 100 µL of derivatization reagent (Meth-Prep™ II, Grace Davison, Columbia, MD, USA), allowing it to react for 30 min at room temperature to posterior gas chromatography-mass spectrometry (GC-MS) analysis. A 5 µL aliquot was injected into the gas chromatograph with helium as the mobile phase at a flow rate of 0.5 mL/min, measured at 150 °C at the top of the column. The Agilent Model 6890 Gas Chromatograph (Agilent Technologies, Santa Clara, CA, USA) was coupled to an Agilent Model 5975 Electron Shock Mass Detector (Agilent Technologies, Santa Clara, CA, USA). The chromatographic column was a Supelcowax^®^ 10 Capillary GC column, 30 m × 0.53 mm, df 0.50 µm (Supelco, Bellefonte, PA, USA). The temperature ramp was started at 150 °C with a temperature gradient from 4 °C/min to 260 °C and after an isothermal temperature was maintained for 15 min. The injector was at 280 ° C. The identification of compounds was carried out by comparison with NIST05 library and all match factors (certainty, %) were higher than 80%.

Resolvin D1 was determined in the hydroalcoholic extract of 1 g of PL via ELISA kit determination (Cayman, Ann Arbor, MI, USA), and coefficient of variation intra-assay 11.4%.

#### 4.6.3. Immune Cell Isolation

PBMCs and neutrophils were obtained from venous blood from 8 voluntary adult healthy subjects. Venous blood samples were obtained from the antecubital vein using suitable vacutainers, with EDTA as anticoagulant. Venous blood samples were obtained after 12 h overnight, under fasted conditions. The PBMCs fraction was purified from whole blood following an adaptation of the method previously described [47] using the Ficoll-Paque PLUS reagent (GE Healthcare^®^, Chicago, IL, USA). The precipitate containing the erythrocytes and neutrophils was incubated at 4 °C with 0.15 M ammonium chloride to hemolyze the erythrocytes. The suspension was centrifuged at 750× *g*, at 4 °C for 15 min, and the supernatant was then discarded. The neutrophil phase at the bottom was washed first with ammonium chloride and then with phosphate buffer saline (PBS), pH 7.4.

#### 4.6.4. MTT Assay

Cell viability was evaluated by a MTT assay, following the previously described method [48]. Concentrations of 0, 0.001, 0.005, 0.01, 0.02, 0.05, and 0.1 mg/mL of 5-dodecanolide were tested to evaluate its toxicity.

#### 4.6.5. Immune Cell Incubations

Incubations of both PBMCs and neutrophils were carried out at 2 × 10^6^ cells/mL in RPMI 1640 culture media containing 2 mM l-glutamine and activated by addition of LPS from *Escherichia coli* (1 μg/mL). The anti-inflammatory properties of the PL hydroalcoholic extract were analyzed in isolated PBMCs and neutrophils stimulated with LPS. Four different treatments were applied: Control group cells treated only with culture medium (RPMI 1640); LPS group: cells treated with culture medium (RPMI 1640) in addition to LPS (LPS 1 μg/mL); PL hydroalcoholic extract group: cells treated with culture medium (RPMI 1640) in addition to PL hydroalcoholic extract at 1 mg/mL. PL hydroalcoholic extract group + LPS: cells treated with culture medium (RPMI 1640) in addition to PL hydroalcoholic extract at 1 mg/mL and 30 min after the incubation began, and LPS at 1 μg/mL was added to the culture media.

The anti-inflammatory properties of 5-dodecanolide were analyzed in isolated neutrophils stimulated with LPS in the presence or absence of the different concentrations of 5-dodecanolide. The capability of neutrophils to produce TNFα was used as marker of inflammation. Neutrophils incubated in RPMI 1640 media (2 × 10^6^ cells/mL) without any treatment were used as a negative control, whereas cells stimulated with LPS (1 μg/mL) to induce an inflammatory response were used as a positive control. The 5-dodecanolide (Quimigen, Spain) at a final concentration of 0.01 mg/mL, 0.06 mg/mL, and 0.1 mg/mL was used to evaluate their influence on TNFα production.

The antioxidant and anti-inflammatory properties of the compounds present in the hydroalcoholic extract were assayed using the ex vivo neutrophil model. The isolated neutrophils were distributed between the following groups: Control (RPMI 1640 media, 2 × 10^6^ cells/mL); LPS (RPMI 1640 media, 2 × 10^6^ cells/mL, LPS 1 μg/mL); LPS + ResolvinD1 (RPMI 1640 media, 2 × 10^6^ cells/mL, LPS 1 μg/mL, Resolvin D 11 ng/mL); LPS + Oleamide (RPMI 1640 media, 2 × 10^6^ cells/mL, LPS 1 μg/mL, Oleamide 0.06 mg/mL); LPS + 5-Dodecanolide (RPMI 1640 media, 2 × 10^6^ cells/mL, LPS 1 μg/mL,5-dodecanolide 0,04 mg/mL); LPS + extract (RPMI 1640 media, 2 × 10^6^ cells/mL, LPS 1 μg/mL, PL hydroalcoholic extract 1 mg/mL). All groups were incubated in polypropylene tubes at 37 °C for 2 h. Subsequently, the cells were pelleted by centrifugation (900× *g* for 5 min at 4 °C) and cell-free supernatants were used to determine the CAT and MPO enzyme activities, as well as cytokine levels.

#### 4.6.6. Cytokine Assays

Pro- and anti-inflammatory cytokines were determined in free cell supernatants. IL-6, IL-8, TNF-α and PGE-1 were measured in culture medium supernatant using ELISA kits. The TNF-α ELISA kit (Diaclone, France) intra-assay and inter-assay reproducibility were 3.3% and 9.0%, respectively. The IL-6 ELISA kit (Diaclone, Besancon, France) intra-assay and inter-assay reproducibility were 4.4% and 9.1%, respectively. The IL-8 ELISA kit (RayBio, Atlanta, GA, USA) intra-assay and inter-assay reproducibility were 10% and 12%, respectively. PGE1 was measured with ELISA (Enzo Life Sciences^®^, Farmingdale, USA). The intra-assay and inter-assay reproducibility was lower than 10% and 12%, respectively.

#### 4.6.7. Statistical Analysis

Statistical analysis was carried out using a statistical package (SPSS 25.0 for Windows^®^, Amonk, NY, USA). The normal distribution of the data was assessed with the Shapiro–Wilk test. The statistical significance was determined by one-way analysis of variance (ANOVA). When significant differences were found between groups, a LSD post hoc test was carried out. A probability level (P) of significance of *p* < 0.05 was considered statistically significant. All values in figures and tables are expressed as mean ± S.E.M.

## 5. Conclusions

In conclusion, it was evidenced that PL exerts a remarkable anti-inflammatory effect, whether used as an ointment or as a hydroalcoholic extract. This effect is partially attributable to the presence of 5-dodecanolide, although the effects of this compound alone do not reach the magnitude of the anti-inflammatory effect observed by the hydroalcoholic extract. We propose that the presence in the hydroalcoholic extract of other compounds, such as oleamide, hexadecanoic acid, 9-octadecenoic acid, 6-octadecenoic acid, 9,12-octadecadienoic acid, octadecanoic acid, 5,8,11,14-eicosatetraenoic acid, 7-hexadecenoic acid, 11,14-octadecadienoic acid, hexadecanamide, and resolvin D1, increases the anti-inflammatory potential of 5-dodecanolide.

## Figures and Tables

**Table 1:** Effects of pig lard treatment on antioxidant, pro-inflammatory, and oxidative damage markers in inflamed plantar tissues and neutrophils.

	Saline Buffer + Vaseline	Saline Buffer + Pig Lard	Zymosan + Vaseline	Zymosan + Pig Lard
**Enzymatic activities in subplantar tissue**				
**MPO** (nKat/mg protein)	2.79 ± 0.30	3.02 ± 0.28	21.8 ± 2.41 ^a^	17.8 ± 3.02 ^a^
**GPx** (nKat/mg protein)	0.29 ± 0.02	0.32 ± 0.04	0.38 ± 0.02	0.36 ± 0.06
**CAT** (K/mg protein)	11.3 ± 2.81	12.9 ± 1.23	9.17 ± 1.02	10.7 ± 2.23
**Enzymatic activities in neutrophils**				
**MPO** (nKat/μg DNA)	4.47 ± 0.82 ^a^	4.74 ± 0.44 ^a^	7.73 ± 1.03 ^b^	4.44 ± 0.67 ^a^
**GPx** (nKat/μg DNA)	0.80 ± 0.06	0.79 ± 0.06	0.87 ± 0.02	0.64 ± 0.04
**CAT** (K/mg DNA)	0.15 ± 0.02 ^a^	0.14 ± 0.01 ^a^	0.27 ± 0.06 ^b^	0.16 ± 0.03 ^a^
**Oxidative damage markers**				
**MDA** (nmol/mg protein)	523 ± 79.3	542 ± 86.6	648 ± 48.4	591 ± 79.2
**Carbonyls** (μmol/mg protein)	15.1 ± 1.13	16.7 ± 1.73	23.9 ± 0.92 ^a^	24.2 ± 1.13 ^a^
**Nitrite** (μmol/mg protein)	5.81 ± 1.06	6.04 ± 0.54	14.1 ± 1.02 ^a^	11.6 ± 0.75 ^b^

Statistical analysis: One-way ANOVA test. Different letters indicate significant differences (*p* < 0.05).

**Table 2:** Pig lard hydro alcoholic extract composition.

Molecule (Certainty%)	Percentage (%)
Hexadecanoic (93%)	0.603 ± 0.09
9-Octadecenoic (99%)	61.6 ± 2.21
6-Octadecenoic (99%)	13.3 ± 0.83
9,12-Octadecenoic (99%)	0.31 ± 0.17
5,8,11,14-Eicosatetraenoic (90%)	5.77 ± 0.42
5-Dodecanolide (81%)	4.49 ± 0.15
7-hexadecenoic (89%)	2.08 ± 0.72
11,14-Octadecadienoic (93%)	5.84 ± 0.15
Hexadecanamide (92%)	1.07 ± 0.21
Octadecenemide (82%)	5.02 ± 0.52
Resolvin D1 (μg/g of extract)	2.86 ± 0.28

Results are % on molar basis. Resolvin D1 is expressed as μg/g of extract.

## Data Availability

Not applicable.

## Sample Availability

Not available.
